# Brain Hypertrophy in Patients With Mesial Temporal Lobe Epilepsy With Hippocampal Sclerosis and Its Clinical Correlates

**DOI:** 10.1212/WNL.0000000000210182

**Published:** 2024-12-23

**Authors:** Richard Zubal, Matus Velicky Buecheler, Daichi Sone, Tjardo Postma, Jane De Tisi, Lorenzo Caciagli, Gavin P. Winston, Meneka K. Sidhu, Lili Long, Bo Xiao, Andrew William Mcevoy, Anna Miserocchi, Sjoerd B. Vos, Christian R. Baumann, John S. Duncan, Matthias J. Koepp, Marian Galovic

**Affiliations:** 1 Department of Neuroradiology, Inselspital, Bern University Hospital and University of Bern, Switzerland;; 2 Department of Neurology, Clinical Neuroscience Center, University Hospital and University of Zurich, Zurich, Switzerland;; 3 Department of Psychiatry, Jikei University School of Medicine, Tokyo, Japan;; 4 Department of Clinical and Experimental Epilepsy, UCL Queen Square Institute of Neurology, London, United Kingdom;; 5 MRI Unit, Epilepsy Society, Chalfont St Peter, Buckinghamshire, United Kingdom;; 6 Department of Psychiatry, Amsterdam UMC, Vrije Universiteit Amsterdam, Amsterdam, the Netherlands;; 7 Department of Bioengineering, University of Pennsylvania, Philadelphia, PA;; 8 Division of Neurology, Department of Medicine, Queen's University, Canada;; 9 Centre for Neuroscience Studies, Queen's University, Canada;; 10 Department of Neurology, Xiangya Hospital, Central South University, Changsha, China;; 11 Centre for Medical Image Computing, Department of Computer Science, University College London, United Kingdom; and; 12 Centre for Microscopy, Characterisation, and Analysis, The University of Western Australia, Nedlands, Australia.

## Abstract

**Background and Objectives:**

Mesial temporal lobe epilepsy (mTLE) is generally associated with focal brain atrophy, but little knowledge exists on possible disease-related hypertrophy of brain structures. We hypothesized that repeated seizures or adaptive plasticity may lead to focal brain hypertrophy and aimed to investigate associated clinical correlates.

**Methods:**

In this cohort study, we included patients with mTLE undergoing detailed epilepsy evaluations and matched healthy volunteers (HVs) from 2 tertiary centers (discovery and validation cohorts). We assessed areas of brain hypertrophy and their clinical correlates using whole-brain voxel-based or surface-based morphometry (VBM, SBM), subcortical volumetry, and shape analysis of T1-weighted MRI data by fitting linear models. We evaluated the functional implications of the findings on memory encoding using fMRI.

**Results:**

We included 135 patients with mTLE with neuropathology-confirmed hippocampal sclerosis (77 left, 58 right; 82 women; mean age 37 ± 11 years) and 47 HVs (29 women, mean age 36 ± 11 years) in the discovery cohort. VBM detected increased gray matter volume of the contralateral amygdala in patients with both left (*t* = 8.7, *p* < 0.001) and right (*t* = 7.9, *p* < 0.001) mTLE. We confirmed the larger volume of the contralateral amygdala using volumetry (left mTLE 1.74 ± 0.16 mL vs HVs 1.64 ± 0.11, *p* < 0.001; right mTLE 1.79 ± 0.18 mL vs HVs 1.70 ± 0.11, *p* = 0.002) and shape analysis (left mTLE *p* ≤ 0.005; right mTLE *p* = 0.006). We validated the hypertrophy of the contralateral amygdala in the validation cohort (mTLE, n = 18, 1.91 ± 0.20 mL; HVs, n = 18, 1.75 ± 0.13; *p* = 0.009). In left mTLE, contralateral amygdala hypertrophy was associated with poorer verbal memory and, in right mTLE, with more frequent focal-to-bilateral tonic-clonic seizures. A larger volume of the contralateral amygdala correlated with increased functional activation of the right parietal memory encoding network in a subgroup (44/135 patients with mTLE, 26/47 HVs) receiving fMRI.

**Discussion:**

Unilateral mTLE due to hippocampal sclerosis is associated with hypertrophy of the contralateral amygdala. This may represent plasticity to compensate for verbal memory deficits or may be the consequence of seizure spread to the contralateral hemisphere.

## Introduction

Mesial temporal lobe epilepsy (mTLE) is a common focal epilepsy syndrome in adults.^1^ A common cause of mTLE is hippocampal sclerosis.^2^ Many imaging studies show that atrophy in mTLE is not restricted to the hippocampus but affects widespread and bilateral networks of cortical and subcortical structures^3^ and may be progressive during the course of the disease.^4,5^

Hypertrophy, on the contrary, has received little attention in mTLE. Animal studies showed that repeated kindled seizures were associated with a thickening of the CA1 hippocampal subregion.^6^ Seizure-induced hypertrophy was also observed in the amygdala, dentate gyrus, and piriform cortex and may be related to expansion of the neuropil due to swelling of astrocytes.^6-8^ In humans, increased volume of the amygdala, hippocampus, and parahippocampal gyrus was observed in individuals with sudden unexpected death in epilepsy (SUDEP) and those at high risk of SUDEP.^9^ This hypertrophy was mainly observed in those with a high frequency of convulsive seizures and may, thus, be a seizure-related phenomenon.^10^ Hypertrophy of the amygdala, piriform cortex, and parahippocampal gyrus was also observed in people with frontal lobe epilepsy.^11^ Bilateral amygdala enlargement was observed in patients with depression^12^ and in psychosis of epilepsy.^13,14^

Hypertrophy may also be related to adaptive physiologic neuronal plasticity. Well-known examples include hippocampal hypertrophy reflecting spatial navigation training in London taxi drivers^15^ and volume increase of posterior temporoparietal areas in volunteers who learned to juggle.^16^

We tested the hypothesis that the effects of repeated seizures or adaptive plasticity may lead to focal hypertrophy in people with mTLE and hippocampal sclerosis and that this would have clinical correlates.

## Methods

In this study, we analyzed the magnitude, localization, and determinants of focal hypertrophy using several objective neuroimaging methods and validated the findings in an external cohort.

### Discovery Cohort

Data were collected as part of an ongoing single-center prospective cohort study of long-term outcomes after epilepsy surgery that includes all people receiving resective epilepsy surgery at the National Hospital for Neurology and Neurosurgery in London, United Kingdom.^17^ In this study, we included participants with unilateral mTLE and pathology-confirmed hippocampal sclerosis who had a preoperative MRI scan on the same 3T MRI GE Signa HDx scanner using a coronal T1-weighted 3D inversion-recovery fast spoiled gradient-echo sequence (0.9 × 0.9 × 1.1 mm voxel dimensions; repetition time/echo time/inversion time = 8.1/3.1/450 ms). Diagnosis of TLE was made by a multidisciplinary epilepsy team based on clinical history, neurologic examination, seizure semiology, MRI, and neuropsychological and psychiatric assessments. Hippocampal sclerosis was confirmed in all cases by neuropathology after surgery.

We compared the patients with an age-matched and sex-matched comparison group of healthy volunteers (HVs) who did not have a history of epileptic seizures and neuropsychiatric or genetic disorders and presented with a normal neurologic examination and structural MRI.^18,19^

### Validation Cohort

We validated our main volumetric results in an independent previously published external cohort.^2^ The inclusion criteria for patients were the diagnosis of sporadic TLE with an asymptomatic sibling. All participants had an MRI scan on the same 3T MRI GE Signa HDx scanner using a coronal T1-weighted magnetization-prepared rapid gradient-echo sequence (1 × 1 × 1 mm voxel dimensions; repetition time/echo time/inversion time = 7,792/2.98/800 ms). Participants were recruited at the Xiangya Hospital, Central South University, Hunan Province, China.

### Standard Protocol Approvals, Registrations, and Patient Consents

The discovery cohort data set comprised deidentified previously collected routine clinical data without the need for individual consent, as approved by the UK Health Research Authority (22/SC/0016). Healthy volunteer data were acquired as part of 2 previous studies,^18,19^ and all HVs provided written informed consent.

In the validation cohort, the ethics committee of Xiangya Hospital approved the study and all participants provided written informed consent.

### Voxel-Based and Surface-Based Morphometry

Gray matter volume, total intracranial volume, and cortical thickness were estimated using the fully automated^20^ and validated^21^ Computational Anatomy Toolbox CAT12,^22^ in Statistical Parametric Mapping 12 (SPM12).^23^ Gray matter was segmented using Unified Segmentation, normalized using Diffeomorphic Anatomical Registration Through Exponentiated Lie Algebra, and smoothed with a 12-mm volume-based kernel. Cortical thickness was estimated using the projection-based thickness method followed by topology correction, spherical mapping, spherical registration, and smoothing with a 15-mm surface-based kernel. Data were inspected visually using the retrospective quality assurance protocol implemented in CAT12.

### Subcortical Segmentation and Volumetry

We used a parcellation algorithm based on Geodesic Information Flows available within NiftyWeb^24^ to segment the amygdala. For hippocampal segmentation, we used Hipposeg,^25^ an algorithm specifically developed to segment the hippocampus in people with epilepsy with no more variability than seen between expert human raters.^26^ Next, a blinded rater reviewed the anonymized masks of the amygdala and the hippocampus and corrected any incorrect segmentations. The blinded rater was guided by anatomical landmarks^27^ and previously established protocols for manual segmentation.^28^ We have previously shown that such a combined semiautomated procedure yielded a high intra-rater (0.98 ± 0.01) [mean ± SD] and inter-rater (0.96 ± 0.02) reliability.^29^

### Subcortical Shape Analysis

We converted the segmentations of the amygdala and hippocampus to 3-dimensional surface meshes and parametrized these with a spherical harmonic point distribution model (SPHARM-PDM).^30^ Minimal smoothing of the edges was necessary to ensure a spherical morphology of the segmentations. We generated a mean mesh template from 40 HVs and aligned all surface meshes to this mean mesh. We visually inspected all shapes for both surface mesh and alignment failures. Displacement values were generated using a point-to-mesh approach calculating the distance between the mean template surface and each point on an individual's surface mesh.

### fMRI Memory Encoding

As a post hoc analysis, we evaluated the functional impact of amygdala hypertrophy that was detected using the volumetric methods described above. To validate our hypothesis that adaptive plasticity may lead to focal hypertrophy, we assessed the relationship of amygdala hypertrophy with activation patterns on a verbal memory encoding fMRI paradigm. We postulated this based on the association of amygdala hypertrophy with verbal learning scores in our primary analyses and also in light of our previous findings,^31,32^ which showed critical engagement of both ipsilateral and contralateral mesial temporal lobe structures for verbal and visual memory encoding in patients with TLE.

We evaluated data from a previously published fMRI study.^31^ Details of the cohort and data acquisition have been described previously and are summarized in eMethods.

In brief, the verbal memory encoding paradigm involved the presentation of concrete nouns to participants during a single scanning session. Participants were told to memorize these for an out-of-scanner word recognition task. In the recognition task, participants were asked to classify recognized items as remembered, familiar (if unsure), or novel using a button box. These responses were used to sort each stimulus seen in the scanner to successfully remembered, familiar, or forgotten. Recognition accuracy (%) was calculated (% true positive—% false positive) and used as a regressor of no interest as mentioned further (further details in eMethods). Regressor of interest for words was formed by creating a box-car function for words convolved with the canonical hemodynamic response function. Movement parameters were included as confounders, and parameter estimates for the regressors were calculated for each voxel. Contrasts were generated for words corresponding to the main effect of the task. Individual word encoding contrast images were used for the second-level analysis.

We analyzed the correlation between verbal memory fMRI encoding in the combined group of left and right TLE and volume of the contralateral amygdala using linear regression, correcting for age, sex, total intracranial volume, and task performance (postscan recognition accuracy). We performed sensitivity analyses assessing left and right TLE separately (eAppendix 1 and eFigures 1–5). We report fMRI results at a *p* < 0.05 on a cluster level corrected for multiple testing using family-wise error (FWE) correction.

### Statistical Analysis

We corrected the volumes of the final segmentations for the effects of age and total intracranial volume using an equation derived from linear regression estimated in the healthy control data set. We compared the corrected volumes, participant demographics, and clinical data using the χ^2^ test for categorical variables and independent-sample *t* test for continuous variables. The amygdala volumes of patients with TLE in the validation cohort were flipped to reflect ipsilateral or contralateral amygdalae and to increase statistical power in this smaller cohort. A similar number of healthy control amygdalae in the validation cohort were also flipped. We assessed the association of corrected volumetric data with clinical variables using linear regression. Data are presented numerically as N (%) or mean ± SD. Calculations were performed in the SPSS statistical analysis package, version 25.0 (IBM-SPSS, Armonk, NY).

We compared the morphometric imaging data (voxel-wise gray matter volume, vertex-wise cortical thickness, point-based surface shape displacement) using fixed-effect linear models adjusting for age, sex, and total intracranial volume. We report morphometric results at a *p* < 0.05 threshold corrected for multiple comparisons using random field theory.

## Results

We included 135 patients with unilateral TLE (77 left, 58 right) and 47 HVs who were comparable for age (*t* = −0.6, *p* = 0.52) and sex (odds ratio 1.0, *p* = 1.00). Baseline characteristics are presented in the Table.

**Table:** Baseline Characteristics

	LTLE n = 77	RTLE n = 58	Controls n = 47
Sex, n (%)			
Female	44 (57)	38 (66)	29 (62)
Male	33 (43)	20 (34)	18 (38)
Age (y)			
Age at scan	37 ± 11	38 ± 10	36 ± 11
Age at seizure onset	13 ± 10	11 ± 8	N/A
Duration of epilepsy	25 ± 14	26 ± 13	N/A
Handedness			
Right	57/74 (77)	50/55 (91)	N/A
Left	17/74 (23)	5/55 (9)	N/A
Seizures			
FASs	46/74 (62)	20/55 (36)	N/A
FIASs	72/74 (97)	53/55 (96)	N/A
FBTCSs	61/74 (82)	44/55 (80)	N/A
Number of AEDs, n (%)			
None	1 (1)	0	47 (100)
1	6 (8)	1 (2)	0
2	33 (43)	26 (45)	0
3	31 (40)	25 (43)	0
4	6 (8)	6 (10)	0
Neuropsychometry			
Verbal learning^a^	−1.22 ± 0.95	−0.79 ± 0.94	N/A
Visual learning^a^	−0.62 ± 1.27	−0.86 ± 1.00	N/A
Psychosis	8/74 (11)	3/55 (5)	N/A
Volumes (cm^3^)			
Right amygdala	1.74 ± 0.16	1.61 ± 0.21	1.64 ± 0.11
Left amygdala	1.66 ± 0.21	1.79 ± 0.18	1.70 ± 0.11
Right hippocampus	2.94 ± 0.27	1.91 ± 0.40	2.83 ± 0.22
Left hippocampus	1.91 ± 0.38	2.82 ± 0.31	2.80 ± 0.23

Abbreviations: FASs = focal aware seizures; FBTCSs = focal-to-bilateral tonic-clonic seizures; FIASs = focal seizures with impaired awareness.

Data are shown as N (%) or mean ± SD.

a z-scores.

### Gray Matter Hypertrophy

**Figure 1:**
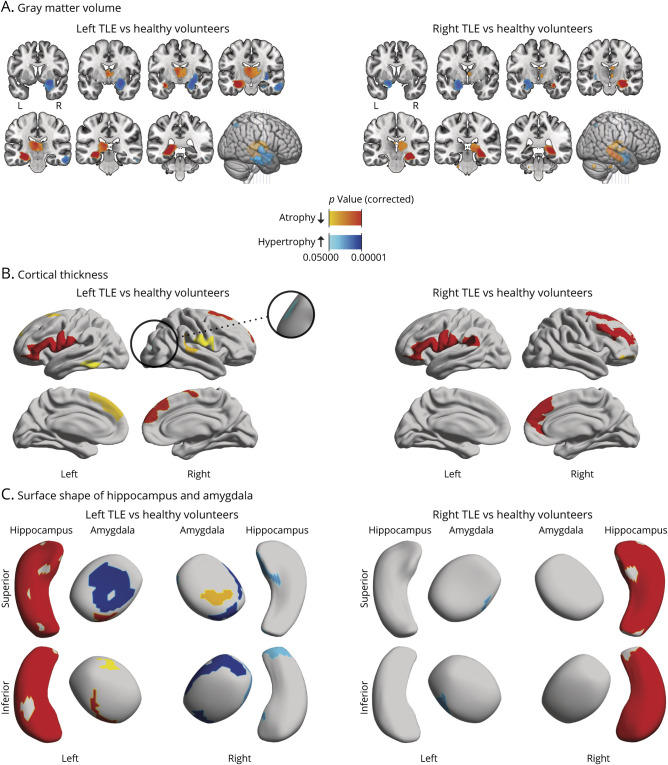
Morphological Analyses of Atrophy and Hypertrophy in mTLE Panel A shows changes in gray matter volume assessed using voxel-based morphometry. The location of the coronal slices is displayed on a 3D brain model (bottom right). Panel B shows changes in cortical thickness. Panel C shows subregional morphological changes of the hippocampus and amygdala assessed using surface shape analysis. Atrophy is displayed using the yellow-red color scheme, and hypertrophy is shown in blue. All results were thresholded at *p* < 0.05 on a cluster level after correction for multiple testing.

Whole-brain voxel-based morphometry detected areas of gray matter hypertrophy in the contralateral mesial temporal lobe with a local maximum over the contralateral amygdala and extending toward the contralateral putamen in left (*t* = 7.7, *p* < 0.001) and right (*t* = 7.9, *p* < 0.001) TLE (Figure 1A, blue color for hypertrophy). In addition, there were small areas of focal hypertrophy in the contralateral middle temporal gyrus (*t* = 6.6, *p* < 0.001), ipsilateral angular gyrus (*t* = 5.9, *p* = 0.001), and ipsilateral frontal piriform cortex (*t* = 5.4, *p* = 0.01) in left TLE and the contralateral superior parietal lobule (*t* = 5.4, *p* = 0.004) in right TLE. The full voxel-based morphometry results including the findings for gray matter atrophy are displayed in eAppendix 2 and eFigures 6–9.

### Cortical Thickening

Surface-based morphometry detected small areas of focal cortical thickening (Figure 1B, blue color for hypertrophy) in the contralateral precentral gyrus (2.9 resels, *p* = 0.005) and the contralateral cuneus (2.2 resels, *p* = 0.03) in left TLE. We did not detect any cortical thickening in patients with right TLE compared with HVs. Cortical thinning is presented in eAppendix 3 and eFigures 10 and 11.

### Volumetric Data

**Figure 2:**
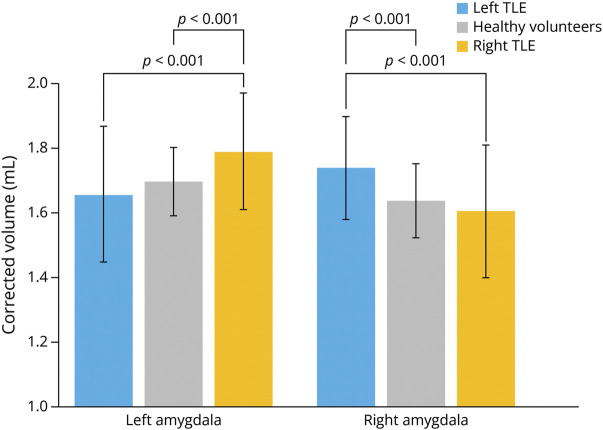
Volumetry of the Amygdala The figure shows amygdala volumes in left mTLE (blue), HVs (gray), and right mTLE (orange). Vertical lines represent standard deviations. HVs = healthy volunteers; mTLE = mesial temporal lobe epilepsy.

The volumes of the amygdalae are displayed in Figure 2. The volume of the contralateral amygdala was larger in patients with left (mean 1.74 ± 0.16 mL, *t* = 3.8, *p* < 0.001) and right (mean 1.79 ± 0.18 mL, *t* = 3.2, *p* = 0.002) TLE compared with HVs (mean left amygdala 1.70 ± 0.11 mL; right amygdala 1.64 ± 0.11 mL). Ipsilateral amygdala volumes in patients with TLE did not differ from HVs.

In left TLE, a larger contralateral amygdala was associated with longer duration of epilepsy (β = 0.004 per year, *p* = 0.002), worse verbal learning scores (β = −0.04 per one SD, *p* = 0.046), and a history of psychosis (β = 0.14, *p* = 0.02). In right TLE, a larger contralateral amygdala was associated with more frequent focal-to-bilateral tonic-clonic seizures (FBTCSs) (β = 0.07 per seizure per month, *p* = 0.002). Full correlations are provided in eTable 1.

We also observed a larger contralateral hippocampus in patients with left TLE (mean 2.94 ± 0.27 mL, t = 2.5, *p* = 0.02) but not in patients with right TLE (mean 2.82 ± 0.31 mL, t = 0.4, *p* = 0.68) compared with HVs. Details on hippocampal atrophy are shown in eAppendix 4.

### Amygdala Surface Shape

Amygdala surface shape analysis showed focal hypertrophy of the contralateral (7.5 resels, *p* < 0.001) and ipsilateral (5.5 resels, *p* < 0.001) amygdala in left TLE. In right TLE, we found small areas of focal hypertrophy in the contralateral amygdala (2.4 resels, *p* = 0.006). There were small areas of focal atrophy affecting the ipsilateral (cluster 1, 3.5 resels, *p* < 0.001; cluster 2, 1.7 resels, *p* = 0.03) and contralateral (2.1 resels, *p* = 0.01) amygdala in left TLE (Figure 1C). There were no areas of focal atrophy of the amygdala in right TLE.

### Hippocampal Surface Shape

Hippocampal surface shape analysis showed small areas of hypertrophy in the contralateral medial hippocampal head (2.4 resels, *p* = 0.005) and hippocampal tail (2.1 resels, *p* = 0.01) in left TLE. There was no hypertrophy in right TLE. There was pronounced atrophy affecting the ipsilateral hippocampus in patients with left (45.9 resels, *p* < 0.001) and right (44.5 resels, *p* < 0.001) TLE compared with HVs (Figure 1C).

### Validation Cohort

The validation cohort included 18 patients with unilateral sporadic TLE (12 right, 6 left), their 18 asymptomatic full siblings, and 18 matched HVs. We replicated the hypertrophy of the contralateral amygdala in patients with mTLE (n = 18, mean 1.91 ± 0.20 mL) compared with HVs (n = 18, mean 1.75 ± 0.13 mL, *p* = 0.009) in the validation cohort. Unaffected siblings of people with mTLE had a normal volume of the amygdala (n = 18, 1.76 ± 0.12 mL, *p* = 0.84).

### Memory fMRI

**Figure 3:**
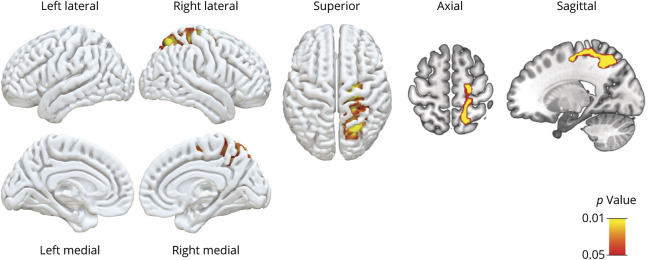
Association of Amygdala Hypertrophy With Altered Activation of Memory Networks This figure displays the correlation between volume of the contralateral amygdala and fMRI memory encoding activation in the right superior parietal lobe obtained using a verbal encoding task. FMRI results are reported at a *p* < 0.05 on a cluster level corrected for multiple testing using family-wise error correction.

As a post hoc analysis, we evaluated the association of amygdala hypertrophy with fMRI memory encoding activation patterns in a previously published cohort of 44 patients with unilateral mTLE and hippocampal sclerosis and 26 healthy controls. Larger volume of the contralateral amygdala correlated with increased activation of areas in the right superior parietal lobe (Figure 3, 943 voxels, t = 4.58, *p* = 0.01 FWE corrected) during verbal memory encoding in the combined patient group. Activations within these regions were not associated with task performance. The full fMRI results are given in eAppendix 1. Group memory encoding results in HVs and patients with left and right TLE have been published previously.^31^

### Data Availability

Anonymized data that these results were based on and were not published within this article will be made available on request from any qualified investigator.

## Discussion

We assessed focal hypertrophy in a well-characterized cohort of patients with mTLE and pathologically confirmed hippocampal sclerosis. We demonstrated hypertrophy of the contralateral amygdala in both left and right TLE groups using several methodologies (voxel-based morphometry, volumetry, surface shape analysis). We replicated this result in an independent external cohort. Hypertrophy of the contralateral amygdala correlated with longer duration of epilepsy and worse verbal learning in left mTLE and with more frequent FBTCSs in right mTLE. Hypertrophy of the contralateral amygdala was associated with altered activation of the memory encoding network in TLE. There were several other small cortical and subcortical areas that showed less pronounced effects, but the findings in these areas were not robust across methods or epilepsy lateralization.

Our results consistently demonstrated hypertrophy of the contralateral amygdala across different methods in both left and right mTLE groups and in a validation cohort. A previous study focusing on morphology of the amygdala in mTLE also found occasional hypertrophy of the amygdala that was 93% contralateral to the epileptic focus.^33^ The largest morphological study in epilepsy published by the ENIGMA-Epilepsy Working Group^34^ reported volumes of the amygdala in the online supplement. There was significant hypertrophy of the contralateral amygdala in left (Cohen *d* 0.27, *p* = 0.009) and right (Cohen *d* 0.23, *p* = 0.01) mTLE after correction for multiple testing using false discovery rates. Taken together, several well-powered studies found consistent evidence for hypertrophy of the contralateral amygdala in mTLE.

A number of previous studies focused on hypertrophy of the *ipsilateral* amygdala.^35-37^ In these cases, hypertrophy was believed to be the cause rather than the consequence of epilepsy. A neuropathologic analysis demonstrated that, in most of these cases, ipsilateral hypertrophy was caused by dysplastic lesions or low-grade tumors.

In contrast to ipsilateral hypertrophy, contralateral hypertrophy of the amygdala is unlikely to be the cause of seizures. All of our patients were deemed to have unilateral mTLE arising from the opposite temporal lobe after detailed presurgical evaluation. Comprehensive presurgical evaluation in these cases did not reveal concerns of contralateral or bilateral epileptic foci. The contralateral amygdala appeared normal on visual analysis, and hypertrophy was only detected using quantitative methods on a group level. Thus, an underlying contralateral tumor or dysplastic lesion is unlikely.

Consequently, hypertrophy of the contralateral amygdala may be a consequence of unilateral mTLE. It was not observed in unaffected full siblings of people with TLE in the validation cohort. This indicates that genetic or familial factors are unlikely to contribute to contralateral amygdala hypertrophy. It may, thus, represent a phenomenon acquired during the course of the disease. In support of this, hypertrophy of the contralateral amygdala was related to longer duration of left mTLE.

One possible explanation for hypertrophy of the contralateral amygdala is compensatory plasticity due to structural and functional deficits of the ipsilateral mesial temporal lobe. This is supported by the observation that hypertrophy of the right amygdala was associated with worse verbal learning in those with left mTLE, in a usually language-dominant hemisphere. We hypothesized that hypertrophy of the right amygdala could represent inefficient compensation of verbal memory deficits. Our fMRI findings support this hypothesis as we showed that larger amygdala volume correlated with parietal activations. We also observed that these activations did not correlate with task performance, possibly suggesting inefficient reorganization. We previously showed that reorganization of the verbal memory network to parietal regions was not as efficient as reorganization to mesial temporal structures, insula, and temporal neocortex in both left and right TLE.^31,38^

We did not find an association of amygdala hypertrophy with visual learning. This could be due to the more bilateral representation of visual learning compared with verbal learning.^29^ Thus, visual memory deficits may be more easily compensated and may be less prone to cause compensatory hypertrophy.

An alternative explanation for hypertrophy of the contralateral amygdala is the harmful effect of seizures. In this regard, we observed an association of amygdala hypertrophy with a higher frequency of FBTCSs in right mTLE. Increased volume of the amygdala was previously observed in a cohort of people at high risk of sudden unexpected death in epilepsy, who are also more likely to have more frequent FBTCSs.^9^ Similarly, our recent study reported larger amygdala volumes and disrupted architecture bilaterally in people with epilepsy and FBTCS, especially in the presence of postconvulsive central apnea.^10^ Seizures may cause reactive gliosis, thickening of the neuropil, and inflammation.^8,39^ Seizures that propagate to both cerebral hemispheres were more likely to affect the contralateral amygdala.

Hypertrophy assessed with voxel-based morphometry was more widespread in patients with left TLE compared with right TLE. This could be related to the effects on language networks in the left, usually language-dominant, hemisphere that may have caused larger network effects affecting the contralateral hemisphere. Another possible explanation is a bigger statistical power in left mTLE due to a bigger sample size. In left mTLE, shape analysis also detected areas with an outward deviation in the ipsilateral amygdala while volumetry did not show changes in the overall volume of the ipsilateral amygdala. Thus, this observation could point to a deformation rather than hypertrophy of the ipsilateral amygdala related to shift of brain structures after atrophy of the hippocampus.

We also found an association of a larger contralateral amygdala in left mTLE with a history of psychosis. This has been reported previously, and our data confirm these results.^13^ Although the explanation for this finding is unknown, one study proposed increased inhibition or decreased excitation of amygdala nuclei as a potential mechanism leading to psychotic symptoms.^40^ We did not find an association of contralateral amygdala volume with a history of depression or anxiety.

Our study has limitations. First, our results only apply to mTLE with hippocampal sclerosis, and we did not assess other epilepsy types. Second, a limitation inherent to most epilepsy studies is the potential impact of antiseizure medications on the results. However, amygdala volume did not correlate with the number of antiseizure medications (eTable 1), thus making a relevant impact on the results less likely. Third, we did not have data on the time interval between the last seizure and MRI; thus, we cannot correlate the impact of very recent seizures on hypertrophy. Last, we did not quantitatively analyze T2 or FLAIR imaging in our study, but there were no abnormalities reported in the contralateral hemisphere on visual analysis in the included cases.

To conclude, unilateral mTLE due to hippocampal sclerosis is associated with hypertrophy of the contralateral amygdala. Our results suggest that this hypertrophy may represent plasticity to compensate for verbal memory deficits or may be the consequence of seizure spread to the contralateral hemisphere. These findings reinforce the concept of epilepsy as a network disorder and highlight the impact of unilateral epilepsy on the contralateral hemisphere.

## Glossary

FWE: family-wise error
HVs: healthy volunteers
mTLE: mesial temporal lobe epilepsy
SBM: surface-based morphometry
SUDEP: sudden unexpected death in epilepsy
VBM: voxel-based morphometry
